# Plant Identity Influences Decomposition through More Than One Mechanism

**DOI:** 10.1371/journal.pone.0023702

**Published:** 2011-08-16

**Authors:** Jennie R. McLaren, Roy Turkington

**Affiliations:** Department of Botany and Biodiversity Research Centre, University of British Columbia, Vancouver, British Columbia, Canada; Duke University, United States of America

## Abstract

Plant litter decomposition is a critical ecosystem process representing a major pathway for carbon flux, but little is known about how it is affected by changes in plant composition and diversity. Single plant functional groups (graminoids, legumes, non-leguminous forbs) were removed from a grassland in northern Canada to examine the impacts of functional group identity on decomposition. Removals were conducted within two different environmental contexts (fertilization and fungicide application) to examine the context-dependency of these identity effects. We examined two different mechanisms by which the loss of plant functional groups may impact decomposition: effects of the living plant community on the decomposition microenvironment, and changes in the species composition of the decomposing litter, as well as the interaction between these mechanisms. We show that the identity of the plant functional group removed affects decomposition through both mechanisms. Removal of both graminoids and forbs slowed decomposition through changes in the decomposition microenvironment. We found non-additive effects of litter mixing, with both the direction and identity of the functional group responsible depending on year; in 2004 graminoids positively influenced decomposition whereas in 2006 forbs negatively influenced decomposition rate. Although these two mechanisms act independently, their effects may be additive if both mechanisms are considered simultaneously. It is essential to understand the variety of mechanisms through which even a single ecosystem property is affected if we are to predict the future consequences of biodiversity loss.

## Introduction

Despite increasing responses to biodiversity loss (such as an increase in protected areas) resulting from the 2002 Convention on Biological Diversity, the rate of biodiversity loss does not appear to be slowing [1]. Certain species, or groups of species, are characterized by traits that make them more likely to decrease in abundance, or become locally extinct, under certain environmental drivers [2], and changes in biodiversity are necessarily accompanied by changes in species composition. For example, nitrogen deposition often benefits grasses, but results in a decrease in forb abundance [3]. As plant functional groups influence a variety of ecosystem properties differently [4], [5], changes in species composition are likely to affect ecosystem functioning.

Changing the types or number of plant species in a community may affect decomposition rates through at least two mechanisms. First, different species or functional groups of plants have varying effects on many ecosystem properties [4], [5] and consequently changes in the plant community may affect the local decomposition microenvironment such as soil temperature [6], the decomposer community [7] and competition for nutrients between the vegetation and the saprotrophic community [8].

Second, changing the members of the living plant community necessarily changes its contribution to the composition and quality of the litter community. Individual species vary in their decomposition rates [9] because of differing leaf characteristics such as leaf nitrogen and lignin contents [10], carbon quality [11] and secondary chemicals [12], [13]. Litter is rarely composed of a single species, and the combination of litter from multiple species may also affect decomposition rate. Although litter mixing studies have produced no consistent patterns relating litter diversity to decomposition rates (reviewed by [14]), numerous studies have reported non-additive effects of mixing different litter types, where litters decompose at different rates in mixture than they do in monoculture [15]–[17].

Finally, interactions between these two mechanisms also may affect decomposition rate and experiments that compare their relative impacts are rare. A few studies have examined these two mechanisms independently, including examination of the effects of tree species identity [6], and plant species diversity [18]–[20]. However, inconsistent results from these studies may result both from a lack of strong diversity effects [18] and from experimental designs that did not allow a test of interactions between the two mechanisms [6]. More recently, three studies placed different litter combinations into plant communities having different levels of richness or species composition and this allowed a direct examination of interactions [21]–[23]; all three studies reported interactions between the two mechanisms.

In addition, plant identity effects on decomposition, through one or both mechanisms, may be dependent on environmental conditions, or context. Although changes in plant composition are likely to be accompanied (or caused) by changes in environmental conditions, few studies examine plant identity effects on decomposition in more than one environmental context. We examine effects of plant functional group identity on decomposition within two different environmental contexts to examine constancy of effects across both soil fertility levels and changes in abundance of mycorrhizae. Both of these environmental changes are relevant to this northern ecosystem; global warming is expected to cause an increase in soil nutrient levels, especially in northern latitudes, because higher temperatures increase mineralization rates of both nitrogen and phosphorus [24], [25]. This increase in nutrients, and other environmental changes, are expected to influence both the composition and the functioning of the soil mycorrhizal community [26]. Both environmental factors are also likely to influence litter decomposition directly. Rates of litter decomposition are generally thought to be limited by the availability of nitrogen because of the inverse relationship between C:N and decomposition and because N accumulates in litter during early decay [27], but not all studies support this conclusion -- even in N-limited systems [28]. Mycorrhizal fungi in the soil also may directly affect litter decomposition rates, as mycorrhizae may have saprotrophic functions [29]. They could also act indirectly, because a decrease in mycorrhizal colonization may reduce the ability of plants to compete with saprophytic soil microflora [30].

In this study we examined the effects of plant functional group identity on decomposition through both changes in the decomposition microenvironment and changes in the species composition of the litter. We removed a single plant functional group from a series of plots in a grassland community in northern Canada. By comparing plots that had functional groups removed with control plots having an entire community of species, we could determine the role of functional group identity on decomposition in the intact community. We examined the effects of different plant functional groups in different environments by crossing the removal treatments with a fertilizer and a fungicide treatment (used to decrease mycorrhizal colonization rates). In a previous study we examined the effects of removals on decomposition of a single grass species [31]. Here, we expand on this previous study to include litter from multiple functional groups in our examination of effects through the decomposition microenvironment, and to examine effects of changes in litter species composition. To examine litter composition effects, we created a series of litter bags with all possible combinations of leaves from the dominant species of each the three functional groups and these bags were placed in all three environments (removals, fertilization and fungicide).

This experimental design has three advantages over previous studies. First, by placing all litter combinations in all environments, our design permitted us to investigate the interactions between changes in the environment and changes in litter composition. More uniquely, because we were able to distinguish species within mixtures after decomposition, we could test changes in species-specific decomposition as causes for non-additive effects in mixtures. Finally, we repeated this experiment in two separate years to examine the consistency of results across time. Many field experiments are run in only a single location, or for a single growing-season, and a significant result gives the expectation that, were the experiment to be repeated in a different location or year, the results would be the same. Of the seven studies already described that examined effects of richness or composition on litter decomposition through both mechanisms [6], [21]–[23]; none repeated their examination of the effects of litter composition on decomposition rates, and only one [20] examined environmental effects on decomposition in more than one year.

## Materials and Methods

This removal experiment was part of a larger experiment examining the role of plant functional group identity in determining various ecosystem functions. McLaren & Turkington [5], describe the methods in detail, and they are described briefly below.

### Site Description

The study area is a dry grassland near Kluane Lake in the south-western Yukon in northern Canada (61°04.218 N 138°23.018 W). The area receives a mean annual precipitation of ca. 230 mm, about half of which falls as rain during the summer, but also includes an average annual snowfall of about 100 cm. The grassland is surrounded by a closed to relatively open spruce forest community dominated by *Picea glauca* (Moench) Voss. The grassland is dominated by *Poa glauca* Vahl and *Carex stenophylla* Wahlenb. ssp. *eleocharis* (Bailey) Hultén, and also contains many non-leguminous forbs (dominated by *Artemisia frigida* Willd., *Erigeron caespitosus* Nutt.), and legumes (dominated by *Oxytropis campestris* (L.) DC.); all nomenclature follows Cody [32]. Grassland species were divided into three functional groups, namely, graminoids (grasses and sedges), forbs, and legumes.

### Experimental Plant Communities

Experimental plots were established in May 2003 and maintained annually for 4 years through the 2006 growing season. The experiment was a 4×2×2 fully crossed factorial design (4 removal treatments, +/0 fertilizer, +/0 fungicide). Each of the 16 treatments was replicated 5 times, resulting in a total of 80 plots.

There were four removal treatments: independent removal of each of the three functional groups (graminoids, forbs and legumes) and a no-removal control. Functional groups were chosen based on traits that were potentially relevant to the ecosystem properties of interest (e.g. C:N, stature, N-fixation ability). In 2003, plants were removed from the plots using Round-up™ glyphosate, a non-selective herbicide. Herbicide was painted precisely to the leaves and once plants had visibly yellowed, stems of selected plants were clipped at soil level and removed from the plots. Removal treatments were maintained in 2004 using herbicide application and clipping, and in the subsequent two years the very minimal regrowth was clipped at ground level early in the growing season, but other functional groups were allowed to invade the newly available space created by the removals.

Fertilizer and fungicide treatments were applied upon completion of the removals (July 20) in 2003 and in early June of each subsequent year. Fertilizer was applied each year to half the plots in granular form at a rate of 17.5 g N.m^−2^, 5.8 g P.m^−2^ and 5.8 g K.m^−2^. This application rate was used to be consistent with many other studies being done in the area (e.g. [33], [34]). The fungicide Benlate™ (active ingredient benomyl) was applied to half of the plots as a soil drench (2 L.m^−2^ plot) every two weeks from early-June to mid-August at a rate of 2.5 g benomyl.m^−2^ per application. Plots that did not receive fungicide received an equivalent amount of water. Benomyl applications reduced mycorrhizal colonization rates from 50% to less than 10% of root intersections [35]. It has been suggested that benomyl may cause a number of unintended side effects, such as effects on bacterial densities [36], or to non-mycorrhizal fungi in the system. Marshall et al. [37] showed that benomyl application had no effect on total fungal biomass in this system. Further, in the most comprehensive test of benomyl effects to date, Smith, Hartnett and Rice [36] reported that the principal effect of benomyl was suppression of mycorrhizal fungi, and that there were only small effects on other soil properties.

### Decomposition experiment

Fresh leaf material from the dominant species from each functional group - *Poa glauca* (55% of the total graminoid biomass from seven species), *Artemisia frigida* (20% of the total forb biomass from 13 species), *Oxytropis campestris* (92% of the total legume biomass from 3 species) (for species lists see [35]) – was dried at 40 C for 48 hours and placed in 10×5 cm litter bags made from 1 mm mesh size nylon screening. Although a 1 mm mesh size may prevent some invertebrate decomposers from accessing the litter, leaves and leaflets of these species were too small to be retained by a larger mesh size. To preserve the leaf structural properties, leaves were not ground or cut, except *P. glauca*, which was cut into 8 cm lengths to fit into the litterbags. All possible combinations of 1, 2 and 3 species were created using a replacement series design i.e., total leaf biomass per litter bag was held constant at 0.6 g and mixtures were made up of 0.6 g (monocultures), 0.3 g (2 species mixtures) or 0.2 g (3 species mixtures) of each of the component species.

The decomposition experiment was done in 2004 and repeated in 2006. In mid-June each year (shortly after the growing season began), one replicate bag of each of the seven possible species combinations was placed into each plot. Litter bags were placed into gaps in the vegetation, in contact with the litter layer, and secured to the surface. Litter bags were collected in early August, after approx. 50 days, when the plants in the surrounding community had senesced. Decomposed leaves were removed from bags, cleaned of accumulated soil and new plant material, dried at 60 C for a minimum of 48 hours and weighed. We were still able to differentiate between species post-decomposition, and thus for bags with multiple species, species material was separated and dry mass recorded independently for each species.

Although senesced material is often preferable for decomposition studies, we decided to use fresh material for both experiments as a standard substrate to assess the effects of our treatments, rather than mimic natural decomposition processes, as it was easy to collect and sufficient material was available for the number of replicates required. A number of recent decomposition studies have used green litter [15], [38] and a previous study in this system showed that although fresh material decomposed faster, effects of functional group removals on graminoid decomposition were similar for both fresh and senesced plant material [31]. Although this material is not ‘litter’ in the strictest sense, for ease of reading we refer to this process as ‘litter decomposition’ throughout the paper.

### Analysis

Decomposition is expressed as a proportion of dry mass loss occurring during the single growing season in the field. Individual species masses within species combinations were pooled (creating a single decomposition value per bag) for all except species-specific analyses. The proportion decomposed was standardized as:
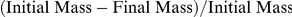



We used a 4-way ANOVA on proportion decomposed with the three environments examined within each year (functional group removal, fertilizer and fungicide) and litter species composition (hereafter termed litter composition) used as main effects. When there was a significant environment X litter composition interaction, analyses were subsequently run independently for each environment level. When the effects of either functional group removals or litter composition were significant, the removals or litter mixtures respectively were analyzed using a Tukey's post-hoc comparison of means.

We calculated expected decomposition of litter mixtures based on monoculture decomposition rates. As all species in a mixture were included in equal proportions, the expected decomposition rate is the average of the monoculture rates for the two (or three) species in the mixture. When monoculture rates differed between environments, expected decomposition was calculated based on environment-specific monoculture rates. Observed to expected comparisons were standardized as:
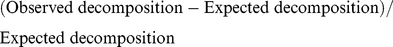



A positive value indicates positive non-additive effects of species mixing on decomposition, and a negative value indicates negative non-additive effects. The mean value of each composition treatment was compared against zero using a t-test.

We analyzed species-specific decomposition within species combinations using a nested ANOVA, with species nested within composition. A Tukey's post-hoc comparison of all means was used to examine species decomposition rates within and between species mixtures. All analyses were conducted using JMP statistical software (2003 SAS Institute, Cary, NC, USA).

## Results

### Effects of decomposition microenvironment

**Figure 1:**
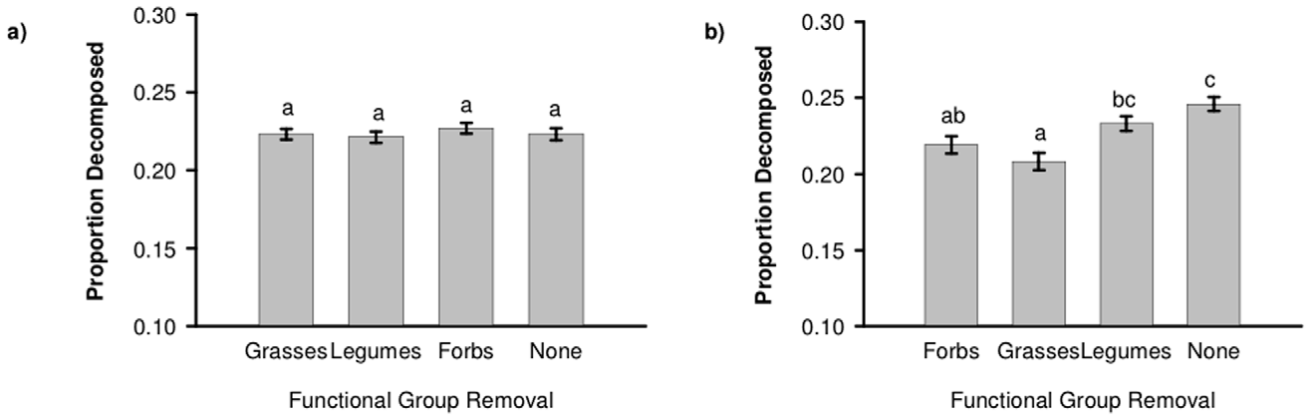
Removal effects on decomposition. Standardized litter decomposition ( (Initial - Final Mass)/Initial Mass) (pooled mean for all litter compositions, ±SE) in different functional group removal treatments in **a)** 2004 and **b)** 2006. Different letters indicate significant differences between removal treatments (p<0.05, Tukey's comparison of all means).

The effects of plant functional group removals on decomposition varied by year; in 2004 removals had no effect on decomposition (Table 1; Fig. 1a) whereas in 2006 both the removal of graminoids and forbs slowed decomposition (Fig. 1b). There was a significant fertilizer x fungicide interaction in both years (Table 1) because in 2004 fungicide slowed decomposition but only in fertilized plots, and in 2006 fertilizer increased decomposition, but only in plots with fungicide. Effect sizes were small in both years.

**Table 1:** Summary of a 4-way ANOVA for the litter decomposition experiment in 2004 and 2006.

Source	df	SS	MS	F	p
Removal	3	0.00	0.00	0.64	0.589
Fungicide	1	0.01	0.01	6.49	0.011
Fertilizer	1	0.00	0.00	0.38	0.536
Composition	6	0.37	0.06	53.86	<0.001
Removal*Fungicide	3	0.00	0.00	1.38	0.247
Removal*Fertilizer	3	0.01	0.00	1.92	0.126
Removal*Composition	18	0.02	0.00	0.78	0.722
Fungicide*Fertilizer	1	0.01	0.01	6.69	0.010
Fungicide * Composition	6	0.01	0.00	0.75	0.608
Fertilizer * Composition	6	0.00	0.00	0.53	0.788
Removal*Fungicide*Fertilizer	3	0.00	0.00	1.10	0.347
Removal * Fungicide * Composition	18	0.03	0.00	1.30	0.180
Removal * Fertilizer * Composition	18	0.01	0.00	0.65	0.860
Fungicide * Fertilizer * Composition	6	0.01	0.00	1.07	0.381
Removal * Fungicide * Fertilizer * Composition	18	0.02	0.00	1.15	0.305

Science Table S1					
		2004		2006	
Source	df	F	p	F	p
Removal	3	0.64	0.589	**13.93**	**<0.001**
Fungicide	1	**6.49**	**0.011**	0.99	0.321
Fertilizer	1	0.38	0.536	0.40	0.530
Litter Composition	6	**53.86**	**<0.001**	**33.65**	**<0.001**
Removal*Fungicide	3	1.38	0.247	0.78	0.507
Removal*Fertilizer	3	1.92	0.126	1.48	0.219
Removal* Litter Composition	18	0.78	0.722	0.49	0.963
Fungicide*Fertilizer	1	**6.69**	**0.010**	**4.79**	**0.029**
Fungicide * Litter Composition	6	0.75	0.608	**2.27**	**0.036**
Fertilizer * Litter Composition	6	0.53	0.788	0.49	0.812
Removal*Fungicide*Fertilizer	3	1.10	0.347	2.46	0.062
Removal * Fungicide * Litter Composition	18	1.30	0.180	0.53	0.946
Removal * Fertilizer * Litter Composition	18	0.65	0.860	0.39	0.989
Fungicide * Fertilizer * Litter Composition	6	1.07	0.381	0.40	0.877
Removal * Fungicide * Fertilizer * Litter Composition	18	1.15	0.305	0.89	0.591

Bold values are significant at p < 0.05.

### Effects of the litter composition

**Figure 2:**
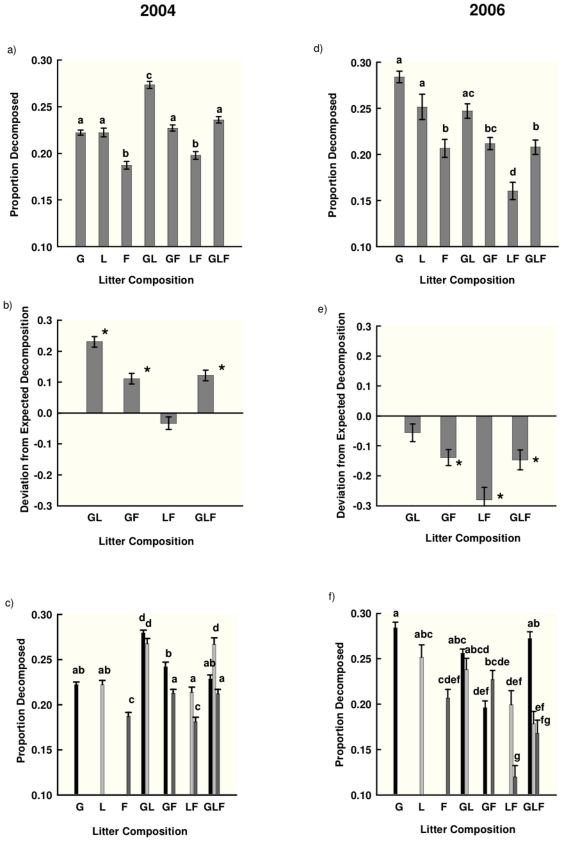
Litter composition effects on decomposition. **a, d)** Standardized litter decomposition (Initial - Final Mass)/Initial Mass) (pooled mean across environments, ±SE) of seven different combinations of species. **b, e)** Standardized deviation from expected decomposition ((Proportion Decomposition Observed - Proportion Expected)/Proportion Expected) (pooled mean across environments, ±SE) of four species combinations. Significant values indicate presence of non-additive effects and an * indicates that a value is significantly different from 0 (t-test, p<0.05). **c, f)** Standardized litter decomposition (Initial - Final Mass)/Initial Mass) (pooled mean across all environments, ±SE) for each species within seven species combinations. Plots from 2004 (a,b,c) and 2006 are separated (d,e,f). For 2006, there were few differences in patterns between non-fungicide and fungicide plots, and only non-fungicide plots are presented for simplicity. For all panels, different letters indicate significant differences between species within litter compositions (Tukey's comparison of all means); G =  graminoid, L =  legume F =  forb.

In 2004, the effect of litter composition, pooled across all environments, was significant (Table 1). Species monocultures decomposed at different rates: the forb (*Artemisia)* decomposed more slowly than either the grass (*Poa*) or the legume (*Oxytropis*) (Fig. 2a). Species mixtures also decomposed at different rates: the grass-legume combination decomposed fastest, while the legume-forb combination decomposed more slowly than all others (Fig. 2a).

Species mixtures displayed positive non-additive effects on decomposition; every combination that contained the grass decomposed significantly faster than expected (GL: t_1,79_ = 13.57 p<0.001, GF: t_1,79_ = 6.58 p<0.001, LF: t_1,79_ = −1.67 p = 0.10, GLF: t_1,79_ = 7.15 p<0.001; Fig. 2b). These non-additive effects can be further explored by examining responses of individual species within each mixture (Litter Composition (Species): F_3,899_ = 21.70, p<0.001) . Within the grass-legume combination, both species had faster decomposition than their respective monocultures (Fig. 2c). In the other two combinations containing grass, the positive non-additive effects were due to an acceleration of the decomposition of the species accompanying the grass in both cases (grass decomposition rates did not differ from monoculture) (Fig. 2c). The legume-forb combination decomposed marginally slower than expected (Fig. 2b), but decomposition rates of neither species within the mixture differed from monoculture (Fig. 2c).

In 2006, the effect of litter composition differed between fungicide treatments (Table 1) and thus litter composition was examined independently within each fungicide treatment (with Fungicide: F_3,279_ = 15.02, p<0.001; without Fungicide: F_3,279_ = 19.63, p<0.001 ). There were few differences in patterns between non-fungicide and fungicide plots, and only non-fungicide plots are presented for simplicity. In both non-fungicide and fungicide plots, the forb decomposed more slowly than the other species (Fig. 2d). Again, species mixtures decomposed at different rates; the grass-legume combination had the fastest decomposition, and the legume-forb combination the slowest in both non-fungicide and fungicide plots (Fig. 2d).

In contrast to 2004, 2006 species mixtures displayed negative non-additive effects on decomposition; every combination that contained the forbs decomposed significantly slower than expected, in both non-fungicide (GL: t_1,39_ = 1.22 p = 0.22, GF: t_1,39_ = −6.01 p<0.001, LF: t_1,39_ = −5.61 p = 0<0.001, GLF: t_1,39_ = −2.32 p = 0.03; Fig. 2e) and fungicide plots (GL: t_1,39_ = −1.91 p = 0.06, GF: t_1,39_ = −5.22 p<0.001, LF: t_1,39_ = −6.71 p = 0<0.001, GLF: t_1,39_ = −4.47 p = 0.03). Again, within the mixtures, species differed in their decomposition rates, in both plots without fungicide (Litter Composition (Species): F_3,479_ = 16.90, p<0.001) and with fungicide(Litter Composition (Species): F_3,479_ = 13.55, p<0.001). In contrast to 2004, there were no clear patterns in species-specific decomposition within mixtures producing these patterns in either non-fungicide (Fig. 2f) or fungicide plots.

## Discussion

In this study we have shown that plant functional group identity affects litter decomposition rates both through effects on the decomposition microenvironment and also through effects on the species composition of the litter. These effects were highly dependent on the year of the study; the presence, direction and species responsible for each of these effects were different in the two years.

### Effects of decomposition microenvironment

Removal of both graminoids and forbs slowed decomposition in one of the two years of this study. This is one of only a few studies to demonstrate significant effects of changing plant community composition on decomposition through changes in the decomposition microenvironment. Previous studies on the effects of the living plant community have produced mixed results with changes in plant species diversity having no strong effect on decomposition [18], [19], [21], but increases in functional group diversity having positive effects [20]. Effects of plant composition on decomposition are more common than the effects of diversity. Effects of species identity on decomposition have been reported in both artificial [6] and natural [22] monocultures of trees, and Jonsson [23] reported that removal of shrubs slowed decomposition. In our earlier study using only a single litter type we also found that both graminoids and forbs in the plant community slowed decomposition, and that this effect was maintained for 5 years [31].

Few studies have been able to characterize which factor in the decomposition microenvironment (resulting from changing plant composition) affects decomposition. Vivanco and Austin [22] measured numerous soil variables, but found nothing that mirrored effects on decomposition rate. Hobbie et al. [6], alternatively, showed tree identity influenced soil temperature, which they speculated influenced decomposition. We measured a variety of soil properties in these removal plots in a previous study [5] but none of the variables measured are likely to result in the observed decomposition patterns. Plots where either legumes or graminoids were removed had similar above-ground biomass in both years, suggesting that biomass effects alone would not drive effects of functional group removal on decomposition. Although removal of forbs and graminoids resulted in similar increases in soil N, and decreases in P [5], as decomposition rate was not directly affected by fertilization, we do not believe decomposition patterns are driven by these differences in soil nutrients. Finally, removal of both graminoids and forbs resulted in higher soil moisture [5], but in this dry ecosystem soil moisture is more likely to encourage rather than retard decomposition [39], [40]. None of these ecosystem properties we examined correspond to changes in decomposition rates, and we suggest that plant identity influences on some other ecosystem property not measured here may be responsible, such as changes in soil temperature as reported by Hobbie et al. [6].

There were few direct effects of any of our other main environmental manipulations (fertilizer and fungicide) on decomposition rates, and interactions between environments were weak and transient. Both treatments were effective as intended; fertilization increased available N, P and K in the soil [5] and fungicide treatments reduced mycorrhizal colonization of roots [37]. Although effects of fertilization on decomposition may be dependent on litter quality [41], [42], we found no strong effects of fertilization despite a large variation in quality of leaf tissue decomposed. Transient decreases in decomposition due to fungicide may be a result of a decrease in mycorrhizal fungi, which can have direct saprophytic functions [29]. Alternatively, fungicide application may have caused a direct reduction in other saprophytic fungi, although Marshall et al. [37] showed that benomyl application had no effect on total fungal biomass in this system.

Finally, we detected no interaction between the removal treatments and fertilizer or fungicide, indicating that the effects of functional group identity on decomposition are not context dependent. Of the studies that have examined the context dependency of species richness on ecosystem functioning, those done in artificially created communities generally showed context dependency [43]–[47], whereas removal experiments in natural communities have shown context dependence of effects in some [48]–[50] but not all [51] studies.

### Effects of litter composition

Functional group identity also affected decomposition via changes in the composition of the litter in both years. Effects of identity were partially due to differences in decomposition rates between species monocultures, with the grass and legume both decomposing faster than the forb. The faster decomposition of the grass was surprising; grasses are often reported to have slower decomposition because of their higher C:N [16], [18] and in this ecosystem, the dominant grass (*Poa* C (44.9%): N(1.7%)) had a higher C:N than both the forb (*Artemisia* C(45.9%) : N(2.4%)) and the legume (*Oxytropis* C (45.2%): N(3.3%)). Other leaf quality factors have been argued to be more important than N in predicting decomposition, such as P [52], C quality [11] and water soluble content [53], even in systems that are otherwise N-limited [11]. We did not measure any other litter quality index, so it is possible that the decomposition rates of these species were determined by another unmeasured trait. For example, secondary metabolites and anti-herbivory alkaloids in *Artemisia* [54] and *Oxytropis* [55] may slow decomposition.

In addition to differences between decomposition rates of the monocultures, litter combinations showed positive non-additive effects of mixing on decomposition in 2004, and negative non-additive effects in 2006. Positive effects of litter mixing are common and reviews report that more than half of all mixtures result in accelerated decay [14], [17]. Our study is unique in that we examined species-specific decomposition rates within mixtures. The acceleration in decomposition in 2004 in only mixtures containing grass was primarily due to an increase in the decomposition of the species associated with the grass, rather than any change in the decomposition of the grass itself. If one uses monoculture decomposition rate as an index of litter quality, the grass litter was of higher quality than either of the associated species, and thus these results support Seastedt's [56] hypothesis that high quality litter could be expected to increase the decomposition rate of associated litter. Numerous mechanisms have been proposed for such an effect including passive and active (by fungi) translocation of nutrients between litter types [57], altering microenvironment characteristics such as water retention within the litter layer [53], and increases in habitat heterogeneity for decomposers [14].

Negative non-additive effects have been reported much less frequently than positive effects; of the 162 mixtures from approximately 30 studies reviewed by Hättenschwiler et al. [14], only 20% reported negative non-additive effects. In our study in 2006 all effects were negative and decomposition of the mixtures was always slower than expected. Although the switch in direction of non-additive effects between years may have been unexpected, the direction of non-additive effects in litter mixing studies have previously been reported to vary with time [15] and litter composition [16], [58].

In addition to the change in direction of the non-additive mixing effects, the species responsible for the effects also changed. Graminoids were central to the positive effects in 2004, but forbs were central to the negative effects in 2006. Although effects of graminoids on mixtures in 2004 were due to a change in the decomposition rate of associated species, such clear species-specific patterns were not present in 2006. Again using monoculture decomposition rates as an index of litter quality, the forb has the poorest quality. These negative non-additive effects support the corollary of the Seastedt [56] hypothesis, i.e., poor quality litter decreases the decomposition rates of mixtures. Mechanisms proposed for such an effect include high amounts of secondary compounds, such as phenolics, in one of the litters [52], or the increased heterogeneity of litter mixtures may prevent the establishment of the subset of decomposers that do best on each litter type individually [59].

The results of this study must be interpreted in the light of a few caveats. We used fresh, rather than senesced leaf material to assess the effects of our treatments on decomposition. We show in a previous study that effects of functional group removals on the decomposition of a single species of grass were similar for both fresh and senesced graminoid plant material, although fresh material decomposed faster [31]. The difference between live and senesced tissue may be larger for the forb and legume than for the grass, as there may be higher levels of nutrient resorption in these relatively nitrogen rich species. The natural input of green litter into ecosystems (such as debris from leaf chewers) is a minor compared to the input of naturally senesced litter. We used fresh material as a standard substrate to assess treatment affects, rather than to mimic natural decomposition processes, and acknowledge that the quality of the fresh vs. senesced leaf material may affect the results of this experiment. Secondly, the decomposition period for both years of the study was relatively short (a single growing season, ca. 50 days). Quality of the decomposing material changes over time, and environmental effects on early decomposition processes may differ from those on latter decomposition stages. In the study referred to above [31], however, we examined effects of functional group removals on both short- and long-term decomposition for a single grass species, and found that effects after a single growing season persisted for up to 5 years of decomposition.

### Differences between Years

There were differences between the 2 years in the effects of plant identity on decomposition for both mechanisms we examined. Effects of functional group removals on decomposition through the decomposition microenvironment were only detected in 2006, and not in 2004. We suspect that this may be due to the age of the plant community. Of the seven similar studies discussed in the Introduction, four were done in young communities (<4 years old) and only one of these [22] showed a significant effect of the decomposition microenvironment. The remaining 3 studies were done in older communities (>10 years old) and all of these reported effects on the decomposition microenvironment. We detected no effects in 2004 when removals had only taken place the previous summer, but did detect effects in 2006, 3 years after the treatments were imposed.

In addition to changes in decomposition microenvironment effects, both the direction of, and the species responsible for, the non-additive effects of litter composition differed between 2004 and 2006. We believe this to be a real effect because our sample sizes were large and our p-values strongly significant. Of the seven studies we discuss in the introduction that examined effects of richness or composition on litter decomposition, none repeated their test of the effects of litter composition on short-term decomposition rates. Thus, we don't know if such a change in the direction of effects between years is an unexpected or unusual result. Environmental context has previously been reported to affect not only decomposition rate, but also the direction of non-additive effects [23], and differences in environmental conditions between the two years may have resulted in the switch in the direction of non-additive effects. In a related study at these sites, we measured numerous ecosystem properties in each of the plots, including plant biomass, soil moisture, light interception, and 14 different soil nutrients, and although there are small differences in these variables between years (higher soil moisture, Fe and Zn and lower Mn, B, S, and Al in 2002 compared with 2004) [5], none of these variables intuitively relate to differences in decomposition between the two years. Although the importance of plant functional group identity in determining the effects of litter mixing is evident, we can only speculate as to what factor(s) caused the switch in the direction of non-additive effects.

Few previous studies have examined the influence of plant species composition on decomposition through both mechanisms, i.e. changes in the decomposition microenvironment and changes in the composition of the decomposing material (e.g. [6], [18], [19]), and fewer had designs enabling them to examine the interaction between these variables. Our experimental design, placing a replicate of each species mixture in all environments, allowed us to examine these interactions and is one of only four studies we know of to do so [21]–[23]. Litter mixing effects did not depend on the identity of living plants present in the community. Two of the previous studies reported significant interactions between the two mechanisms, with legume decomposition increasing with increasing diversity [21] and affinity effects, enhanced decomposition of the species found in the living plant community [22]. Affinity effects, or home advantage, have also been reported by Ayres [60] for three tree species. We were surprised by the lack of interaction in our study as we hypothesized that if there were strong independent effects of both removals and substrate composition, which we found, then an interaction would also occur.

Although we did not detect direct interactions between the effects of decomposition microenvironment and the composition of the decomposing material, we did find indirect interactions between them, which, although less predictable, may be just as important. When we consider multiple-mechanism effects on the same ecosystem property, we have shown that the sum of these effects may produce unexpected increased effects. For example, the loss of graminoids from this ecosystem caused decreases in decomposition both through changes in the decomposition environment and through the loss of the positive effects of graminoids in litter mixtures. Thus, the effects of losing graminoids from this community would be greater than we might predict based on either mechanism alone. In contrast, the presence of forbs in the living community also had positive effects on decomposition through changes in the microenvironment, but their presence in litter mixtures slowed decomposition, and consequently the effects of losing forbs may be less than we might predict based on each mechanism independently. When considering multiple mechanisms, the effects of species loss may be additive, as in the case of the graminoids, or even change from positive or negative to neutral, as in the forbs, despite a lack of direct interactions between the mechanisms.

In conclusion, predicted changes in species or functional group composition due to anthropogenic influences on natural ecosystems make it essential to understand the importance of identity in determining ecosystem properties. It is not only important to understand how changes in composition will affect ecosystems, but also to consider the variety of mechanisms through which composition may affect a single ecosystem process, and to consider these mechanisms in multiple years. Interactions between these different mechanisms may produce results greater than those predicted by any single pathway alone and therefore examining the different pathways through which species loss may affect ecosystem properties is essential for predicting future consequences of human impacts on biodiversity.
